# Application of direct PCR in rapid rDNA ITS haplotype determination of the hyperparasitic fungus *Sphaeropsis visci* (Botryosphaeriaceae)

**DOI:** 10.1186/2193-1801-3-569

**Published:** 2014-09-30

**Authors:** Ildikó Varga, Péter Poczai, István Cernák, Jaakko Hyvönen

**Affiliations:** Plant Biology, Department of Biosciences, University of Helsinki, PO Box 65, Helsinki, FI-00014 Finland; Potato Research Centre, Centre of Agricultural Sciences, University of Pannonia, Festetics u. 7, Keszthely, H-8360 Hungary; Botanical Museum, University of Helsinki, PO Box 7, Helsinki, FI-00014 Finland

**Keywords:** Biological control, Botryosphaeriaceae, diPCR, European mistletoe (*Viscum album*), Genotyping, Polymerase inhibitors, *Sphaeropsis visci*

## Abstract

**Background:**

The plant pathogenic fungus, *Sphaeropsis visci* a dark-spored species of Botryosphaeriaceae, which causes the leaf spot disease of the European mistletoe (*Viscum album*). This species seems to have potential as a tool for biological control of the hemiparasite. For the rapid detection of *S. visci* haplotypes we tested a direct PCR assay without prior DNA purification. This approach was based on a polymerase enzyme from the crenarchaeon *Sulfolobus solfataricus* engineered by fusion protein technology, which linked the polymerase domain to a sequence non-specific DNA binding protein (Sso7d)*.*

**Findings:**

Most isolates of *Sphaeropsis visci* grouped together in our phylogenetic analyses, indicating that isolates had a previously reported haplotype sequence, which is commonly found in the analyzed Hungarian population. This haplotype was also reported from diseased mistletoe bushes from other European countries. We further identified unique single nucleotide polymorphisms (SNPs) in the ITS region, which were specific to the only well resolved clade in the phylogenetic analysis.

**Conclusions:**

The diPCR approach allowed amplification of ITS rRNA gene directly from small amounts of fungal samples without prior DNA extraction. This simple bioassay in plant disease management enables collection of genomic data from fungal plant pathogen populations.

## Introduction

**Figure 1:**
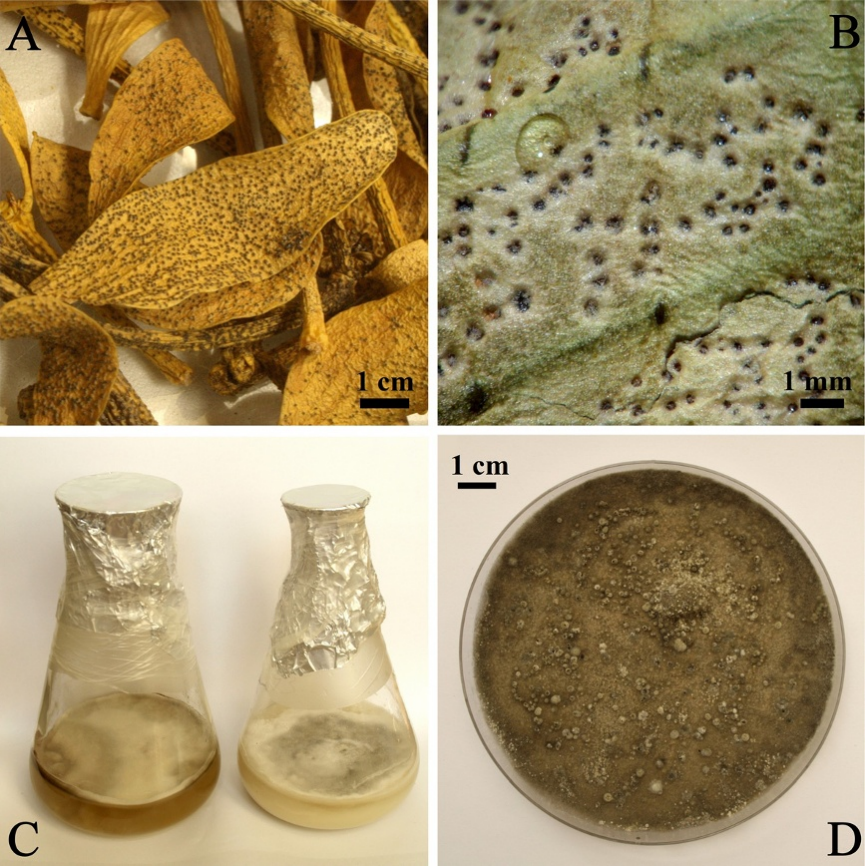
**Leaf spot disease on European mistletoe (** ***Viscum album*** **) caused by** ***Sphaeropsis visci*** **(A, B) and culture characteristics of the fungus (C, D)**. **A**. Symptoms of late infection on: mistletoe leaves and twigs are showing chlorosis. **B**. Symptoms after 20 days of infection; superficial pycnidia are evident on leaves. **C**. *S. visci* liquid culture in potato dextrose (left) and oatmeal broth (right). **D**. *S. visci* culture on oatmeal agar showing dark grey pigmentation.

The hyperparasitic fungal plant pathogen [*Sphaeropsis visci* (Alb. & Schwein.) Sacc*.*], which causes leaf spot disease of European mistletoe (*Viscum album* L.) seems to have potential as a tool for biological control of this hemiparasite (Varga et al. 2012a; Karadžić et al. 2004; Fischl 1996; Stojanović 1989). Three to six weeks after inoculation the fungal infection spreads all over the leaves, branches and berries; a few months later the whole shrub becomes dark yellow and necrotic. *Sphaeropsis visci* (Basionym: *Sphaeria atrovirens* var. *visci* Alb. & Schwein., Consp. fung*.* (Leipzig): 48. 1805. = *Phaeobotryosphaeria visci* (Kalchbr.) A.J.L. Phillips & Crous, Persoonia 21: 47. 2008. For synonyms see (Phillips et al. 2013)) is a dark-spored ascomycete of the family Botryosphaeriaceae (Figure 1). The connection between the asexual and sexual morph of the fungus was established by Phillips et al. (Phillips et al. 2008) by the discovery that the ascomycete *Phaeobotryosphaeria visci* occurring on *Viscum album* produces conidia typical of *Sphaeropsis visci*. Phillips et al. (Phillips et al. 2008) applying a one fungus one name concept chose *Phaeobotryosphaeria* in favor of *Sphaeropsis*. However, the 18^th^ Botanical Congress adopted the Melbourne Code (McNeill et al. 2012) ratifying that priority of names will no longer be based on the life stage of fungi. Thus, the older name *Sphaeropsis* (1880) took priority over *Phaeobotryosphaeria* (1908). The corrections and new name combinations were described in Phillips et al. (Phillips et al. 2013).

The successful control of the European mistletoe would be desirable as it causes considerable damages in many forests and orchards (Baltazár et al. 2013). This causes large economic losses in forestry and agriculture because infected wood becomes unsuitable for processing and infected trees are predisposed to other infections. Mistletoe increases tree mortality and contributes to forest decline (Tsopelas et al. 2004; Idžojtić et al. 2008). With the global warming this might become a more serious problem of larger economic importance. *Viscum album* subspecies do not reach the northern altitudinal limits of their host trees yet (von Tubeuf 1923) as they are temperature-sensitive (Skre 1979; Jeffree and Jeffree 1996). Paleo-climatological studies have used mistletoes as climate indicators (Iversen 1944) as the mean monthly temperatures of the coolest (January) and the warmest month (July) strictly limit the occurrence of *V. album* (Skre 1979). Temperature increase in these months would allow mistletoes to extend their northern latitudinal occurrence. The upper elevation limit of pine mistletoe (*Viscum album* subsp. *austriacum*) in the Rhone Valley of Switzerland rose 200 m in the past century, and during this period the mean winter temperature increased by 1.6°C (Dobbertin et al. 2005). The host plant, *Pinus sylvestris* L., has suffered high mortality in these alpine valleys (Bigler et al. 2006) and pine mistletoe contributed to tree death in this area (Dobbertin and Rigling 2006).

Currently the best method to control mistletoe is to cut off infected branches of the host trees. However, mistletoe cannot be removed completely because it forms adventive shoots from the cortical strands below the cambium of the host (Zuber 2004; Varga et al. 2012b). In order to develop *Sphaeropsis visci* as an effective bio-control agent against *Viscum album* more information is needed about the epidemiology, population biology and the existence of possible haplotypes of this fungus. Fungal biological control agents (BCAs) must perform well in the field tolerating wide range of climatic conditions (fluctuating temperatures, humidities, UV light), edaphic (soil types) and biotic (antagonistic) factors (Butt and Copping 2000). One of the major criticism of fungal BCAs is that they act slowly, therefore factors determining pathogen virulence should be identified and used in strain selection and quality control (Butt and Copping 2000). Cultural conditions must be identified, which retain virulence without increasing production costs. At present little progress has been made in this area with *S. visci*, however, there are efforts to identify optimal growth conditions (Varga et al. 2012c, 2013).

For haplotype determination PCR-based target DNA detection with universal barcode regions is a simple way to identify different haplotypes of plant pathogenic fungi (Queloz et al. 2011; Boose et al. 2011). However, typical PCR protocols require an initial DNA isolation step which is often time-consuming and involves expensive kits or reagents. The isolation of fungal cultures from host plants, again, might be very slow and laborious.

The application of direct PCR (diPCR) allows amplifications without any prior DNA extraction. This is based on recent advances in enzyme technology which has resulted in the increased performance of DNA polymerases *in vitro*. PCR in general tries to mimic the *in vivo* DNA replication, but it is much slower, less efficient, and prone to many errors. Wang et al. (Wang et al. 2004) developed a fusion protein technology by linking the polymerase domain to a sequence non-specific DNA binding protein (Sso7d) from the crenarchaeon *Sulfolobus solfataricus* Zillig et al. (Zillig et al. 1980). This polymerase is reported to lead to a 25-fold lower error rate as compared to common *Thermus aquaticus* Brock & Freeze (*Taq*) polymerase (Wang et al. 2004). Besides a huge increase in efficiency, fusion polymerases are resistant to various PCR inhibitors and have faster overall performance (André 2009). This technology opens possibilities to develop rapid diagnostic methods (e.g., haplotype and species determination) for plant pathogenic fungi by omitting the DNA purification step.

Here we present the application of a diPCR-based assay for the rapid and specific detection of *Sphaeropsis visci* haplotypes without prior DNA purification. The PCR assay amplifies the internal transcribed spacer (ITS) region of the nuclear ribosomal RNA gene. This region is widely used in fungal taxonomy, and used also to identify haplotypes of plant pathogenic fungi (Coates et al. 2002; Kiss et al. 2011; Nechwatal and Mendgen 2009; Bakonyi et al. 2006).

## Methods

### Sample collection

Infected European mistletoe leaves were collected in 2010 and preserved as dry herbarium samples. Sampling was conducted in the western part of Hungary where *Sphaeropsis visci* is commonly found. Sequences from reference strains deposited in MycoBank (http://mycobank.org) and GenBank (Benson et al. 2011) were also included in the study (Table 1).

**Table 1:** **Details about isolates, species and their respective host used in the present study**

Taxon	Sample code	Host	Locality	GenBank accession number	Reference
*Sphaeropsis visci*	Pheo1-20	*Viscum album*	Hungary	JQ291707-JQ291726	This study
*Sphaeropsis visci*	Pheo21-30	*Viscum album*	Hungary	KC759681-KC759690	Varga et al. (2012a)
*Sphaeropsis visci*	CBS186.97	*Viscum album*	Germany	EU673325	Phillips et al. (2008)
*Sphaeropsis visci*	CBS100163	*Viscum album*	Luxembourg	EU673324	Phillips et al. (2008)
*Sphaeropsis visci*	CBS122526	*Viscum album*	Ukraine	EU673326	Phillips et al. (2008)
*Sphaeropsis visci*	CBS122527	*Viscum album*	Ukraine	EU673327	Phillips et al. (2008)
**Outgroups**					
*Sphaeropsis citrigena*	None	*Citrus sinensis*	New Zealand	EU673328	Phillips et al. (2008)
*Sphaeropsis citrigena*	None	*Citrus sinensis*	New Zealand	EU673329	Phillips et al. (2008)
*Phaeobotryon mamane*	None	*Sophora chrysophylla*	Hawaii	EU673331	Phillips et al. (2008)
*Botryosphaeria dothidea*	None	*Prunus* sp.	Switzerland	AY236949	Slippers et al. (2004)
*Diplodia cupressi*	None	*Cupressus sempervierns*	Israel	DQ458893	Alves et al. (2006)

### Direct PCR (diPCR) and sequencing

Excised pycnidia from surface sterilized (1% sodium hypochlorite for 20 min) mistletoe leaves were lysed in 20 μl Dilution Buffer and crushed with pipette tips in Eppendorf tubes. The samples were incubated at room temperature for 5 min; then 0.6 μl of the supernatant was used as template for PCR amplification. PCR was performed with undiluted and diluted DNA extracts from *Sphaeropsis visci* templates including 10 ng to as low as 0.1 pg DNA. DNA was quantified by using a Qubit fluorometer. The amplification was performed with the primers ITS1 (5′-TCCGTAGGTGAACCTGCGG-3′) and ITS4 (5′-TCCTCCGCTTATTGATATGC-3′) designed by White et al. (White et al. 1993) for the fungal Internal Transcribed Spacer (ITS) region of the ribosomal RNA gene. Following the protocol of the Phire Plant Direct PCR Kit, amplification reactions, which were performed in 20 μl volume containing: 7 μl nuclease free water, 0.6 μl sample (from dilution protocol), 0.5 μM of each primer, 10 μl of 2 × Phire Plant PCR buffer and 0.4 μl of Phire Hot Start II DNA Polymerase. All PCR reactions were performed using the following program: 5 min at 98°C for initial denaturation, 40 cycles of 5 s denaturation at 98°C, 5 s annealing at 54°C, and 20 s extension at 72°C, followed by a final extension for 1 min at 72°C. Products were analyzed by gel electrophoresis in 1.5% (w/v) agarose gels (RESolute Wide Range, BIOzym) with 0.5 × TBE electrophoresis buffer (89 mmol/l Tris HCl, 89 mmol/l boric acid, 2 mmol/l EDTA) at 120 V for 1 h and visualized by post-staining with ethidium bromide. The clean-up of PCR products was performed by removing non-incorporated primers with 10 U exonuclease I and degradation of nucleotides by 1 U thermosensitive alkaline phosphatise (Exo I and FastAP, Fermantas, Lithuania). PCR mixes were incubated at 37°C for 15 min and the reaction was stopped by heating the mixture at 85°C for 15 min. Excised fragments were cleaned with NucleoSpin Extract II Kit (Machery-Nagel, Germany). Sequencing was performed in an ABI 3130XL automated sequencer in both directions using the ITS1 and ITS4 primers and the ABI PRISM BigDye Terminator Cycle Sequencing Ready Reaction Kit v.3.0.

### Sequence assembly and alignment

Forward and reverse sequence reads for all isolates were assembled with CodonCode Aligner v.3.7.1. (http://codoncode.org). Discrepancies were manually resolved by editing the traces using the compare option of the advanced assembly function. Single consensus sequences were extracted in FASTA format from the compared assemblies. Multiple sequences were aligned with MUSCLE (Edgar 2004) as implemented in Geneious v.4.8.5 (http://geneious.com) using default settings. Ribosomal exons and spacer regions were annotated in the alignments using the fungal reference sequences deposited in the ITS2 database (http://its2.bioapps.biozentrum.uni-wuerzburg.de/). Obtained sequences were compared with sequences available in GenBank database using the basic local alignment search tool (BLAST). All sequences were annotated and deposited in GenBank (Benson et al. 2011) under accession numbers JQ291707-JQ291726 (Table 1).

### Phylogenetic analysis

Multiple outgroups were included in the phylogenetic analyses based on our previous morphological and molecular identification (Varga et al. 2012a). These were selected from closely and more distantly related clades of Botryosphaeriaceae following Phillips et al. (Phillips et al. 2008). Outgroups included the closely related taxon *S. citrigena* (AJL Phillips, PR Johnst. & Pennycook) AJL Phillips & A Alves, *Phaeobotryon mamane* Crous & AJL Phillips from the sister clade, *Botryosphaeria dothidea* (Moug.) Ces. & De Not. from the more basal branch, and one representative of the distantly related *Diplodia cupressi* AJL Phillips & A Alves from the same family. We supplemented our samples with sequences from other samples of *S. visci* used in our previous study (Varga et al. 2012a).

#### Parsimony analysis

Before making phylogenetic analysis with parsimony as an optimality criterion we were able to reduce the size of the matrix to include only 12 terminals due to numerous, completely identical, duplicates. Due to the small size of the effective matrix we were able to make analysis using the ie command of the program TNT (Goloboff et al. 2008) that ensures finding (all) the most parsimonious tree(s). Jackknife (Farris et al. 1996) support values were calculated using 100 replications with the same search algorithm (ie) as in the search for the parsimonious tree(s).

#### Bayesian analyses

Bayesian analysis was performed with MrBayes v3.2 (Huelsenbeck and Ronquist 2001). The Hasegawa-Kishino-Yano model (HKY) nucleotide substitution model was selected with jModelTest 2 (Darriba et al. 2012) using the Akaike information criterion (AIC) for the dataset. We attempted to sample all trees that have a reasonable probability given the assembled datasets using the Metropolis-coupled Markov chain Monte Carlo (MC)^3^ method. Analyses were initiated with four runs and four chains (8 × 10^6^ generations each). We sampled every 10,000^th^ generation to reduce the size of output files and make the samples more independent. Simulations were run until stationarity was reached assessed according to the average standard deviation of split frequencies < 0.01. MC^3^ convergence was explored by examining the Potential Scale Reduction Factor (PSRF) for all parameters in the model and plots of log-likelihoods over time together with other plots for all parameters allowed by Tracer v1.5 (http://tree.bio.ed.ac.uk/software/tracer/). Additional tests of convergence were conducted with the online program AWTY (Nylander et al. 2008) using the ‘cumulative’ and ‘compare’ functions. The states of the chains sampled before stationarity (split freq. > 0.01) were discarded as burn-in (25%). Trees from BI analyses were summarized as majority-rule consensus trees and edited with TreeGraph2 (Stöver and Müller 2010).

## Results and discussion

**Figure 2:**
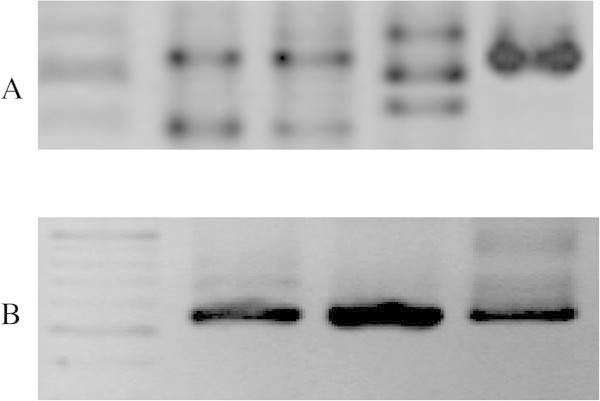
**PCR products from diPCR amplification. A**. Products resulting from fungal contamination. The first line is the size marker indicating 400 to 600 bp range. **B**. Amplified 560 bp size ITS fragments of *Sphaeropsis visci.*

The results show that all tested amounts of template were sufficient to produce amplicons around 560 bp long. However, amplifications with lower concentrations produced only faint bands on agarose gels (data not shown). The analytical sensitivity of diPCR was proved to be high with lower concentrations of DNA that are close (ca. 0.1 pg) to the content of a single fungal cell that is reported to be around ca. 0.15 pg per cell (Kim et al. 2000). This is not surprising as the ITS region is present in multiple copies arranged in tandem repeats in most eukaryotic organisms (Poczai and Hyvönen 2010). In a few cases we detected other fragments of different size in the gel electrophoresis (Figure 2A). All these fragments were excised from the gels and sequenced together with *Spaheoropsis. visci* bands of the expected size of 560 bp. Sequencing and NCBI BLAST search confirmed that products of deviating size belong to the common airborne fungal taxa (Kano et al. 2004) *Cryptococcus magnus* (Lodder & Kreger-van Rij) Baptist & Kurtzman or *Fusarium* sp. with 100% sequence match (sequences not provided but available from the authors upon request). In some cases we also detected larger faint bands (~750 bp) but we were unable to extract them from the gels for sequencing. We anticipate these amplicons were generated from the host (*Viscum album*) as different combinations of the primer set described by White et al. (White et al. 1993) are also used to amplify ITS fragments from European mistletoe (Zuber and Widmer 2000). If additional products are present, fragments showing the expected size should be excised from gels also exemplified by our study. For successful sequencing almost all targeted *S. visci* amplicons were excised from the gels. This requires some additional equipment and time than to simply clean PCR products with exonuclease and phosphatase enzymes. This is contrary to the findings of Aranyi et al. (Aranyi et al. 2014) who used the same approach to identify *formae speciales* (f.sp.) of powdery mildew [*Blumeria graminis* (DC.) Speer]. The lack of plant ITS fragments in their case could be attributed the fact that powdery mildew mycelia could be easily collected from the leaf surface, while pycnidia of *S. visci* are highly embedded in the plant tissues.

We also recommend surface sterilization of the diseased plant leaves and the use of laminar air flow hoods to perform sampling and to set up the PCR when working with plant pathogenic fungi. Direct PCR can be problematic when the contaminant generates amplicons same size as the target region. During our experiments we did not experience such amplicons, but in such cases other laboratory procedures should be applied to obtain satisfactory results e.g. cloning the fragments.

**Figure 3:**
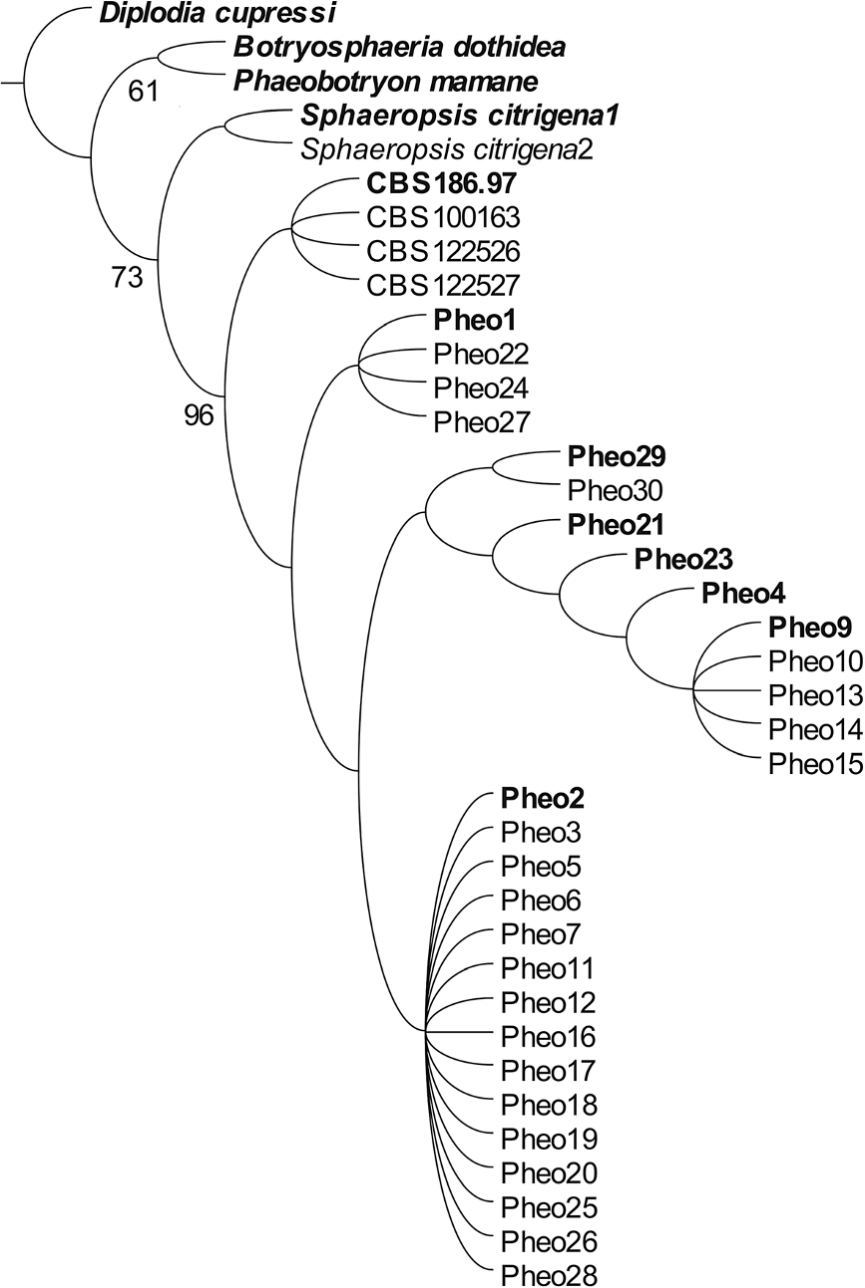
**Most parsimonious tree found by the phylogenetic analysis.** The tree was calculated from 12 (in bold) covering the whole sequence variability. Identical sequences were added to the final tree. Numbers above branches represent jackknife support values.

**Figure 4:**
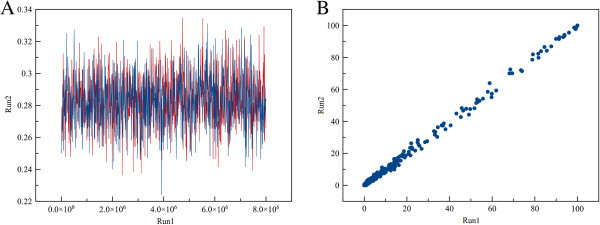
**Examples of output from AWTY.** The plots show the partial graphical exploration of the output from the four different runs of the dataset analyzed in MrBayes v.3.2. **A**. The first graph is the trace plot of the log likelihood (*ln*L) and the sampled values. Blue and red traces indicate run1 and run2, while burn in is not shown on the plots. **B**. AWTY bivariate plot of the split frequencies for comparison between paired Bayesian MCMC simulations from MrBayes analyses. The high correlation shows convergence of the runs.

**Figure 5:**
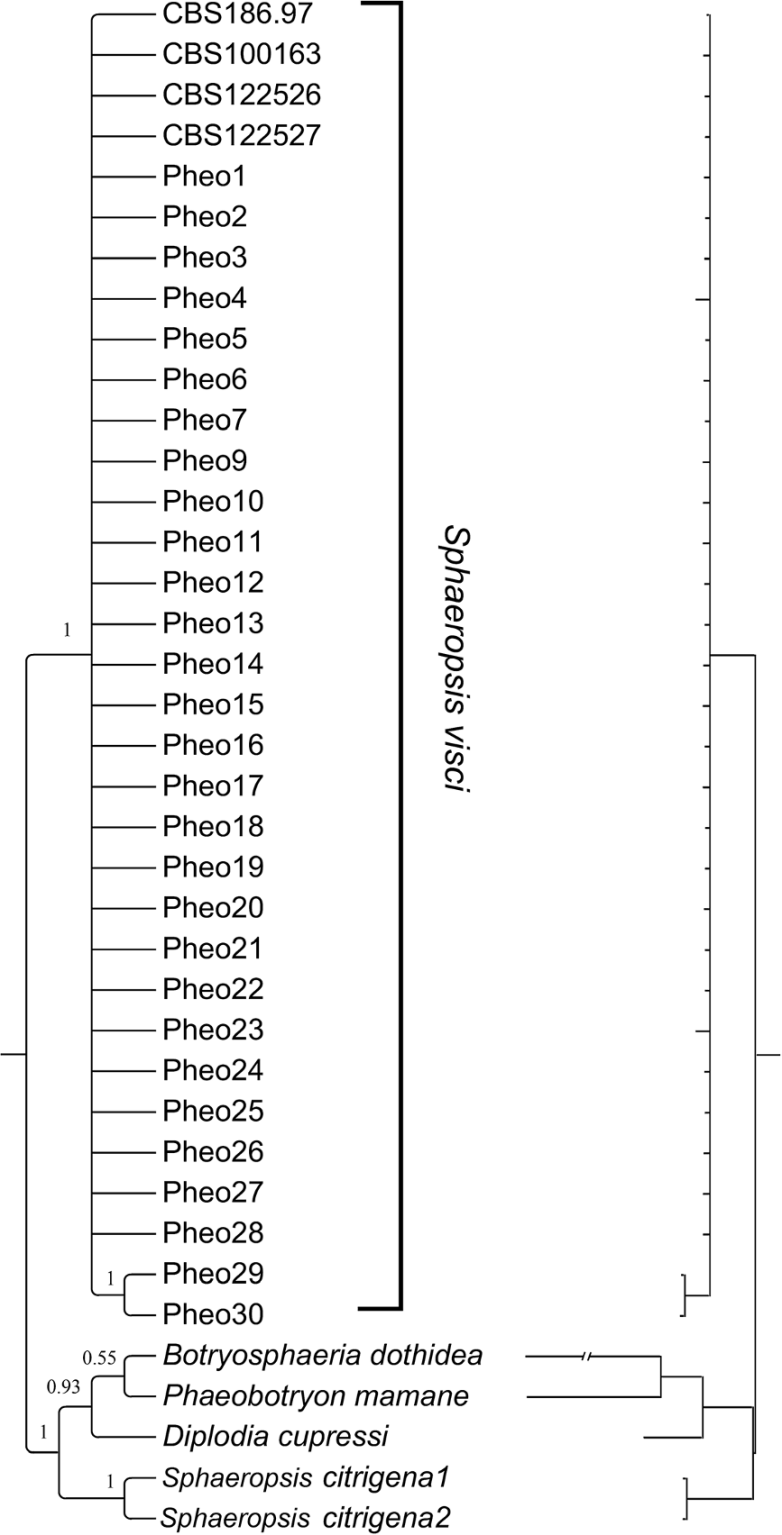
**Bayesian phylogenetic tree.** Majority rule (50%) consensus tree (*left*) and phylogram (*right*) from the analysis of ITS dataset with MrBayes v3.2. Numbers above branches are posterior probabilities (PP).

Parsimony analysis of the matrix of the 12 terminals and 15 parsimony informative characters resulted in one parsimonious tree with a length of 21 steps (Figure 3). Consistency (CI, (Kluge and Farris 1969)) and retention indices (RI, (Farris 1989)) were 0.71 and 0.75, respectively. The results of the two Bayesian runs conducted with MrBayes were highly congruent with each other. PSRF values averaged 1, strongly suggesting that stationarity had been reached. The comparison of the topologies and associated posterior probability values obtained across the independent runs for the dataset with AWTY verified convergence. The inspection of the log likelihood trace plot and the comparison of each run also showed that the runs reached stationarity (Figure 4) after 8 × 10^5^ generations. Both Bayesian and parsimony analyses strongly supported a separate clade composed only by *Sphaeropsis visci* isolates (Figure 5). Branch lengths of the phylogenetic trees indicated a low level of genetic variation among the isolates. Closer inspection of the aligned sequences revealed only a limited number of single nucleotide polymorphisms (SNPs) among the terminals. In the Bayesian analysis internal groups of *S. visci* collapsed on the majority rule consensus tree as the posterior probability of these clades were weak. The detected SNPs were not informative enough to separate them with high posterior probability (PP) values.

A critical trait of pathosystems established in plant populations is the degree to which the same pathogen populations are present and dispersed in the field. The phylogenetic analysis revealed that the majority of the isolates from our study fall in the same group with strains reported in previous studies (Varga et al. 2012a; Phillips et al. 2008). This indicates that there is a widely distributed haplotype that is predominant in *Sphaeropsis visci* populations in Hungary. It has a common ITS sequence matching with sequences known from other countries in Europe (Phillips et al. 2008). *Sphaeropsis visci* is a hyperparasitic pathogen with reported anamorphic reproducing affinity (Phillips et al. 2008). However, we currently know very little about the reproductive biology of this pathogen. Teleomorphs of this species are rarely found and the connection between *Phaeobotryosphaeria visci* and its anamorph *Sphaeropsis visci* was only recently shown (Phillips et al. 2008). The presence of different ITS types in the population could be attributed to sexual recombination as shown by Ahvenniemi et al. (Ahvenniemi et al. 2009) for *Rhizoctonia solani* JG Kühn. However, during our large scale field survey conducted in Hungary during 2010 (Varga et al. 2014) we were unable to find *P. visci* teleomorphs on *Viscum album*. Clonality of this species’ life cycle reduced variation in the analyzed population but this may increase among population, if migration is low. In the absence of high levels of gene flow, local selective sweep will remove variation within populations but will not remove variation between populations (Rydholm et al. 2006). This should be investigated in detail by further large scale analyses sampling further populations of Hungary. Such analysis of the population genetic structure of this hyperparasitic fungus is under way and would be crucial to understand the dynamics of such pathosystems.

## Summary

The diPCR approach presented allows amplification of rDNA ITS bands directly from small amounts of fungal samples without prior DNA extraction. The method reduces the time needed for sample processing and simplifies the workflow for plant pathogenic fungal haplotype analyses. In the traditional ITS analysis DNA from the isolates is obtained by performing, in some cases quite challenging, fungal culturing. Some fungal samples may also contain components which interfere with PCR. As shown here, these culturing steps can be avoided by using the direct PCR method and the problems caused by the presence of PCR inhibitors on the other hand can be avoided by dilution of the template. In summary, the direct PCR method offers a fast and simple bioassay in plant disease management and enables collection of genomic data from fungal plant pathogen without the need to cultivate them.

## Availability and requirements

**Project name:** Hyperparasitic fungal biocontrol agent against European mistletoe.

**Project home page:**http://tuhat.halvi.helsinki.fi/portal/en/person/poczai

**Other requirements:** none

**License:** none

**Any restrictions to use by non-academics:** none
